# Fungal biosynthesis of gold nanoparticles: mechanism and scale up

**DOI:** 10.1111/1751-7915.12151

**Published:** 2014-08-26

**Authors:** Michael Kitching, Meghana Ramani, Enrico Marsili

**Affiliations:** 1 School of Biotechnology, Dublin City UniversityDublin, Dublin 9, Ireland; 2 Marine and Environmental Sensing Technology Hub, Dublin City UniversityDublin, Dublin 9, Ireland; 3 Center for Materials science and Nano Devices, Department of Physics, SRM UniversityKattankulathur, India; 4 Singapore Centre on Environmental Life Sciences Engineering, Nanyang Technological University60 Nanyang Drive, Singapore, 637551, Singapore

## Abstract

Gold nanoparticles (AuNPs) are a widespread research tool because of their oxidation resistance, biocompatibility and stability. Chemical methods for AuNP synthesis often produce toxic residues that raise environmental concern. On the other hand, the biological synthesis of AuNPs in viable microorganisms and their cell-free extracts is an environmentally friendly and low-cost process. In general, fungi tolerate higher metal concentrations than bacteria and secrete abundant extracellular redox proteins to reduce soluble metal ions to their insoluble form and eventually to nanocrystals. Fungi harbour untapped biological diversity and may provide novel metal reductases for metal detoxification and bioreduction. A thorough understanding of the biosynthetic mechanism of AuNPs in fungi is needed to reduce the time of biosynthesis and to scale up the AuNP production process. In this review, we describe the known mechanisms for AuNP biosynthesis in viable fungi and fungal protein extracts and discuss the most suitable bioreactors for industrial AuNP biosynthesis.

## Introduction

In recent years, gold nanoparticles (AuNPs) have gained importance as drug carriers, as their large bioavailable surface permit easy functionalization (Giljohann *et al*., 2010). AuNPs are not only resistant to chemical oxidation, but also stable in a wide range of environmental conditions (Shulka *et al*., 2005; Bhumkar *et al*., 2007; Gong and Mullins, 2009). AuNPs have applications in microbiology and medicine (Storhoff *et al*., 2004; Huang, 2007; Bhattacharya and Mukherjee, 2008; Jelveh and Chithrani, 2011), environmental sensing (Saha *et al*., 2012), and electronics (Huang *et al*., 2003). Examples of AuNP-based technology include biosensors to detect genetic material of bacterial origin (Pissuwan *et al*., 2010), photothermal therapy of tumours (Sperling *et al*., 2008), drug and gene delivery (Ghosh *et al*., 2008; Pissuwan *et al*., 2011) and catalytic removal of environmental pollutants (Narayanan and Sakthivel, 2011b). Interestingly, AuNPs can be synthesized in a variety of shapes, from spheres (Wangoo *et al*., 2008) to rods (Dong and Zhou, 2007) to complex hexagonal crystals (Weng *et al*., 2008). AuNP shape and size determine most of their physical and chemical properties like plasmon resonance (Mock *et al*., 2002), antimicrobial activity (Wani and Ahmad, 2013) and catalysis rate (Zhou *et al*., 2010).

The physicochemical methods for the production of metallic nanoparticles in general and for AuNPs in particular rely either on a top-down or bottom-up approach. A summary of the main pros and cons of these methods is reported in Table 1. In the top-down approach, bulk metal is decomposed into nanoparticles using high laser ablation (Amoruso *et al*., 2014), pyrolysis or attrition (Thakkar *et al*., 2010). These techniques are generally deficient in controlling the nanoparticle size distribution (Mafuné *et al*., 2001) and in maintaining a quality surface structure for the physicochemical and catalytic properties of the AuNPs (Thakkar *et al*., 2010). The production of small AuNPs (diameter < 10 nm) with a more uniform particle size distribution is possible through optimization of the laser pulse time and process temperature (Amoruso *et al*., 2014). However, these techniques employ complex and hi-tech instrumentation facilities, thus involving a high cost production. In the bottom-up approach, AuNPs are constructed atom by atom starting from a precursor gold (Au) salt solution, using either ultrasound (Okitsu *et al*., 2007), radiation (Meyre *et al*., 2008), high temperature (Nakamoto *et al*., 2005), lithography (Wu *et al*., 2014) or chemical/electrochemical methods (Binupriya *et al*., 2010b; Samal *et al*., 2010; Liu *et al*., 2013). Non-polar solvents used in chemical reduction synthesis methods have raised concerns for their environmental toxicity (Narayanan and Sakthivel, 2010). On the other hand, physical methods usually require high temperatures and pressures, which makes it not only difficult to have control over AuNPs size and shape, but also increase energy consumption and production costs. Sol-gel synthesis facilitates the nucleation and growth process of AuNPs, yielding narrower size and shape distribution than those obtained with other physicochemical methods (Kawazu *et al*., 2004). However, sol-gel synthesis requires the use of toxic-capping agents like methyl methacrylate and butyl acrylate to prevent nanoparticle aggregation (Ye *et al*., 2011). Nanoparticles for biomedical application should be prepared only with biocompatible chemicals to minimize their toxic effect and increase their safe usage (Petrucci *et al*., 2014).

**Table 1:** Pros and cons of physicochemical and biological methods for AuNP synthesis

Method	Pros	Cons
Top-down synthesis	Highly controlled particle size distribution and shape.	Extreme conditions, high tech facilities, high cost.
Bottom-up synthesis	Cost-effective. Highly controlled particle size distribution and shape.	Potentially hazardous capping ligands and residual toxins add to environmental toxicity.
Bacteria	Cost-effective and environmentally safe. Biological capping agents for AuNPs stabilization.	Large nanoparticles with broad particle size distribution. It is not possible to obtain pure nanoparticles without any organic components.
Fungi	Cost-effective and environmentally safe. High concentration of extracellular redox enzymes and capping agents for AuNPs stabilization. Smaller size than bacterial-synthesized nanoparticles. Easy scale up.	Broad particle size distribution, low repeatability. It is not possible to obtain pure nanoparticles without any organic components.

Green synthesis through microorganisms might overcome these toxicity issues. Microorganisms like bacteria, algae and fungi synthesize nanomaterials to benefit from their mechanical strength and chemical properties (Das *et al*., 2012a). For example, diatoms mineralize silica to build their cell walls (Frigeri *et al*., 2006), coccolithophore algae mineralize calcium carbonate to form calcite plates and build their exoskeleton (Young *et al*., 1999) and magnetotactic bacteria (e.g. *Magnetospirillum gryphiswaldense*) synthesize magnetite nanoparticles (Schüler, 1999) to enable the bacteria migrate along a magnetic field towards low oxygen environments (Crookes-Goodson *et al*., 2008). Microorganism can synthesize metal nanoparticles through metal bioreduction to remove soluble metals from the surrounding environment, thus decreasing their toxicity and bioavailability. Microorganisms capable of metal bioreduction can colonize metal-contaminated environments. For example, *Shewanella oneidensis* can grow in presence of sub-mM concentration of Ag^+^ (Wang *et al*., 2010), *Geobacter sulfurreducens* can reduce in few hours soluble U^6+^ to insoluble U^4+^ (Orellana *et al*., 2013) and Fe^3+^-reducing mixed cultures can tolerate high concentration of Ni, Cu, Cd, Zn, and Co (Burkhardt *et al*., 2011). A combination of biosorption and bioreduction strategies enable *Aspergillus* sp. isolates from Cr deposits to tolerate up to 1 mM of Cr^6+^ in liquid medium at neutral pH (Fukuda *et al*., 2008). Bioreduction has therefore been extensively studied for its application in the bioremediation of metal-contaminated soil and groundwater. We would like to refer the reader to an excellent review in the area (Lovley, 2003). In microorganisms, extracellular bioreduction reactions might occur in the via microbially produced electron transfer agents (mediated electron transfer) such as the flavins secreted by *Shewanella* sp., which allows the bacteria to shuttle metabolically generated electrons to external electron acceptors (von Canstein *et al*., 2008) or membrane-associated cytochromes and redox proteins (direct electron transfer) (Mukherjee *et al*., 2002; Marshall *et al*., 2008).

The mechanism of metal bioreduction in bacteria, particularly dissimilatory metal-reducing bacteria is now well understood and has contributed to the understanding of nanoparticles biosynthesis in these organisms.

Biosynthetic methods of AuNPs that are based on viable microorganisms or their protein extracts do possess two major benefits: they have a lower environmental impact and increase cost-effectiveness. This is because, for a very low cost, microorganisms produce in a renewable manner both the bioreduction and the capping/stabilizing agents needed in the process, without the need of exogenous chemicals. Furthermore, biosynthetic mechanisms might produce AuNPs of the desired shape, size and distribution given the highly specific interactions of the biomolecular templates and inorganic materials (Cölfen, 2010; Das *et al*., 2012a). The ability to manipulate the shape and size of AuNPs might enable their rational design and functionalization for specific applications (Das and Marsili, 2010; Han *et al*., 2011).

It is worth mentioning here some examples of AuNP biosynthesis of biotechnological relevance in virus, algae and electrochemically active bacteria.

Virus-mediated nanoparticle (VNP) synthesis offer the unique advantage of a size-constrained reaction cage (Douglas and Young, 1998) and displays robust functional groups on the surface of their capsids (Slocik *et al*., 2005). Biosynthesis of nanoparticles in virus finds application in material science and medicine. In general, VNPs are very small, monodispersed, stable and robust, and they might be produced with ease on large scale. Furthermore, VNPs can be modified by either genetic modification of the virus or chemical bioconjugation methods (Steinmetz and Manchester, 2011). Among other applications, viral material could be employed in the reduction of toxic environmental contaminants. For example, reduced iron nanoparticles produced in the filamentous M13 virus reduced soluble uranium (U^6+^) to its insoluble form (U^4+^) (Ling *et al*., 2008).

AuNPs biosynthesis has been reported also in the red alga *Chondrus crispus* and the green alga *Spyrogira insignis* (Castro *et al*., 2013). As dead biomass was used, the bioreduction was likely caused by deprotonated groups on the algal cell wall that act as centres of sorption.

Nanoparticle biosynthesis is essentially a reduction process followed by a stabilization step (capping). Electrochemically active bacteria are capable of metal reduction under a broad range of environmental conditions. Therefore, they can also produce nanoparticles as a by-product of their respiration. For example, washed *Shewanella oneidensis* cells incubated in minimal medium with Au^3+^ produced small spherical AuNPs (see also Table 2) through direct extracellular bioreduction (Anil *et al*., 2011). As this review focuses on AuNPs in fungi, we refer the reader to a recently published review on AuNPs in electroactive microorganisms (Kalathil *et al*., 2013).

**Table 2:** Fungal species capable of AuNPs biosynthesis and location of biosynthetic AuNPs

Species	Reaction conditions	Reaction time (h)	T (°C)	Shape	Size (nm)	AuNP location	Reference
Fungi							
*Alternaria alternate*	Cell-free filtrate	24	RT	Spherical, triangular, hexagonal	12 ± 5		Sarkar *et al*., 2012
*Aspergillus clavatus*	Active biomass	48–72	RT	Triangular, spherical and hexagonal	24.4 ± 11	Extracellular	Verma *et al*., 2011
*Aspergillus niger*	Cell-free filtrate	96	28 ± 2	Spherical, elliptical	12.8 ± 5.6		Bhambure *et al*., 2009
*Aspergillus oryzae var. viridis*	Active and inactive biomass and cell-free extract	72–120	25	Various shapes (cell-free filtrate), mostly spherical (biomass)	10–60	Mycelial surface	Binupriya *et al*., 2010a
*Aspergillus sydowii*	Active biomass	N.A.	N.A.	Spjherical (at 3 mM Au^3+^ concentration	8.7–15.6	Extracellular	Vala, 2014
*Candida albicans*	Cytosolic extract	24	N.A.	Spherical	20–40		Chauhan *et al*., 2011
				Non spherical	60–80		
*Colletotrichum* sp.	Active biomass	96	25–27	Spherical	8–40	Mycelial surface	Shankar *et al*., 2003
				Large aggregates	Undefined		
*Cylindrocladium floridanum*	Active biomass	168	30	Spherical	5–35	Outer surface of the cell wall	Narayanan and Sakthivel, 2011b
*Epicoccum nigrum*	Active biomass	72	27–29	ND	5–50	Intra- and extra-cellular	Sheikhloo *et al*., 2011
*Fusarium oxysporum*	Active biomass	72	N.A.	Spherical, triangular	8–40	Extracellular	Mukherjee *et al*., 2002
*Fusarium semitectum*	Active biomass	24	RT	Spherical	10–80	Extracellular	Sawle *et al*., 2008
*Helminthosporum solani*	Active biomass	72	37 ± 1	Spheres, rods, triangles, pentagons, pyramids, stars	2–70	Extracellular	Kumar *et al*., 2008
*Hormoconis resinae*	Active biomass	24	30	Spherical	3–20	Extracellular	Mishra *et al*., 2010
*Neurospora crassa*	Active biomass	24	28	Spherical	32 (3–100)	Intracellular	Castro-Longoria *et al*., 2011
*Penicillium brevicompactum*	Supernatant, cell-free filtrate, active biomass	12–72	30	Spherical, triangular and hexagonal	10–60	Extracellular	Mishra *et al*., 2011
*Penicillium rugulosum*	Supernatant, cell-free filtrate, and growth medium	8–24	30	Spherical, triangular, hexagonal	20–80		Mishra *et al*., 2012b
				Spherical	20–40		
*Penicillium* sp. 1-208	Cell filtrate	0.08	N.A.	Spherical	30–50		Du *et al*., 2011
	Active biomass	8	N.A.		40–60	Intracellular	
*Rhizopus orzyae*	Cell-free filtrate	24	30	Spherical	16–25		Das *et al*., 2012a
*Saccharomyces cerevisiae*	Active biomass	< 24	30	Spherical	15–20	Cell wall	Sen *et al*., 2011
		> 24			30	Cytoplasm	
*Sclerotium rolfsii*	Cell-free filtrate	N.A.	RT	Spherical	25.2 ± 6.8		Narayanan and Sakthivel, 2011a
*Verticillium* sp.	*Active biomass*	72	28	Spherical	20 ± 8	Cell wall and cytoplasmic membrane	Mukherjee *et al*., 2001
*Volvariella volvacea*	Cell-free extract	N.A.	N.A.	Triangular, spherical, hexagonal	20–150		Philip, 2009
*Yarrowia lipolytica*	Active biomass	120	30	Various shape depending on Au^3+^ concentration	N.A.	Intracellular	Pimprikar *et al*., 2009
Metal-tolerant fungal isolates	Active biomass	24–48	28	Spherical, trigonal, cubic, tetragonal and hexagonal	9–18	Intracellular	Gupta *et al*., 2011
Bacteria							
*Bacillus* *megatherium* D01	Active biomass with dodecanethiol as capping agent	9	26	Spherical	1.9 ± 0.8	Extracellular	Wen *et al*., 2009
Sulfate-reducing bacteria enrichment	Active biomass (high Au concentration)	144	RT	Spherical	< 10	Intracellular and extracellular	Lengke and Southam, 2006
*Escherichia coli*	Active biomass	120	RT	Spherical	25 ± 8	Bacterial surface	Du *et al*., 2007
*Magnetospirillum gryphiswaldense*	Active biomass	1	N.A.	Spherical	10–40	Intracellular	Cai *et al*., 2011
*Marinobacter Pelagius*	Active biomass	22	N.A.	Spherical, triangular	2–10	Extracellular	Sharma *et al*., 2012
*Plectonema boryanum*	Active biomass	24	25	Octahedral	∼ 60	Cell boundary	Lengke *et al*., 2006,2006
*Pseudomonas aeruginosa*	Active biomass	24	37	Spherical	40 ± 10	Extracellular	Husseiny *et al*., 2007
*Rhodobacter Capsulatus*	Active biomass	24	30	Spherical	N.A.	Cell surface and extracellular	Feng *et al*., 2007
*Rhodopseudomonas capsulata*	Active biomass	48	RT	Spherical (pH 7)	10–20	Extracellular	He *et al*., 2007
				Planar (pH 4)	50–400		
*Rhodopseudomonas capsulata*	Cell-free filtrate	48	30	Spherical (low Au^3+^ concentration)	10–20		He *et al*., 2008
				Nanowires (high Au^3+^ concentration)	50–60 (Diameter)		
*Shewanella algae*	Unwashed active biomass	0.5	25	Spherical	10–20	Periplasmic space	Konishi *et al*., 2006
*Shewanella oneidensis*	Active biomass	48	30	Spherical	12 ± 5	Extracellular	Suresh *et al*., 2011

Few references on AuNPs biosynthesis are reported for comparison in the second part of the table. RT, room temperature; N.A., not available.

Many reviews on metallic nanoparticle biosynthesis in bacteria have been already published (Narayanan and Sakthivel, 2010; Thakkar *et al*., 2010). However, only a handful have reviewed the nanoparticle biosynthesis mechanism and attempted to compare the performance of various biosynthetic microorganisms. A good review is available for silver nanoparticle (AgNP) biosynthesis in bacteria, fungi and plants (Durán *et al*., 2011). Another review has described nanoparticles production in fungi but lacked both specific information and mechanistic insight on AuNPs biosynthesis (Dhillon *et al*., 2012).

Current biosynthetic methods have been tested only under laboratory conditions, with somewhat contradictory results and the scale up of biosynthetic processes to industrial applications appears challenging. Despite the theoretical advantage offered by the biosynthetic process over physicochemical processes, recent literature show that slow reaction time, poor reproducibility, insufficient characterization of the Au-reducing proteins, lack of control over the particle size and shape, wide particle size distribution and cumbersome standardization procedures limit the industrial applications of the AuNP biosynthesis process.

In this mini review, we discuss the mechanisms of AuNP biosynthesis in fungal biomass and fungal extract in light of the advantages that fungi offer over other eukaryotes and bacteria and propose possible strategies to overcome the above-mentioned limitations.

## Fungal biosynthesis

While biosynthesis of AuNPs in bacteria is well understood (Rai *et al*., 2013), less than 30 fungal species have been investigated so far for AuNP biosynthesis (Table 2). The occurrence of AuNP biosynthetic capability in bacteria and fungi suggest that the reduction of Au^3+^ to form protein metal nanoconjugates is a common response to toxic stress, where the enzymatic machinery required is readily available in environmental microorganisms.

In general, the microbiology of fungi is much less investigated, mainly because fungi are difficult to characterize – their structure complicates the microscopic and mechanistic studies that are required for nanoparticle characterization in it. Under laboratory conditions, fungi grow at a similar biomass density to bacteria. For example, the biomass yield of a *Rhizopus oryzae* culture grown with glucose as a carbon/energy source in an aerated batch bioreactor was 0.55 g g^−1^ (Taherzadeh *et al*., 2003), whereas an *Escherichia coli* culture grown with glucose in a stirred tank reactor had a biomass yield of 0.31 g g^−1^ (Xu *et al*., 1999).

However, fungi have several advantages over bacteria for bioprocess, including AuNP biosynthesis. Fungi secrete large amounts of extracellular proteins with diverse functions. The so-called secretome include all of the secreted proteins into the extracellular space (Girard *et al*., 2013). The high concentration of the fungal secretome has been used for industrial production of homologous and heterologous proteins. For example, the expression of a functionally active class I fungal hydrophobin from the entomopathogenic fungus *Beauveria bassiana* has been reported (Kirkland and Keyhani, 2011). The tripeptide glutathione is a well-known reducing agent involved in metal reduction and is known to participate in cadmium sulfide (CdS) biosynthesis in yeasts and fungi. Recombinant expression of glutathione in *E. coli* resulted in CdS nanoparticle production (Chen *et al*., 2009). However, the knowledge of the fungal secretome is still at an early stage. The large and relatively unexplored fungal secretome is an advantage because of the role that extracellular proteins and enzymes have in Au reduction and AuNP capping.

Fungal biomass has been used to remove metal cations from water because of the high concentration of cationic biosorption sites (Das, 2010). Particularly at low pH, biosorption on fungal biomass is higher than on bacteria. For example, various Gram-negative bacteria can immobilize Au^3+^ at about 0.35 mM g^−1^ dry cells at pH 3 under non-viable conditions (Tsuruta, 2004). At pH 2.5, *Aspergillus* sp. can immobilize about 1 mM g^−1^ dry cells (Kuyucak and Volesky, 1988).

Based on published literature, it is not possible to compare bacteria and fungi in terms of their metal bioaccumulation ability. In fact, the experimental conditions adopted (e.g. pH, initial metal concentration, temperature) are not homogeneous. Most of the studies are merely descriptive, with little mechanistic information provided. This issue needs to be addressed to develop a general model for AuNP production in viable microorganisms. Repeatable biosynthetic experiments at the same biomass and metal concentration will provide a sound basis to assess metal nanoparticle biosynthesis across bacteria and fungi.

### Mechanism of Au reduction

There are two main precursors of AuNPs in the biosynthetic process: (i) HAuCl_4_, which dissociates to Au^3+^ ions (Khan *et al*., 2013) and (ii) AuCl which dissociates to Au^+^ (Zeng *et al*., 2010). The Au^+^ precursor is much less investigated, likely because of the higher solubility of Au^3+^ ions as compared to Au^+^ ions. However, Au^+^ solubility may be increased through complexation with the appropriate ligands such as alkenes, alkylamines, alkylphosphines, alkanethiols, and halide ions (Zeng *et al*., 2010). While the single-electron reduction of Au^+^ to Au^0^ is comprised of a single step, the three-electron reduction process of Au^3+^ to Au^0^ is likely the combination of a number of chemical transformations (Das *et al*., 2012b).

**Figure 1:**
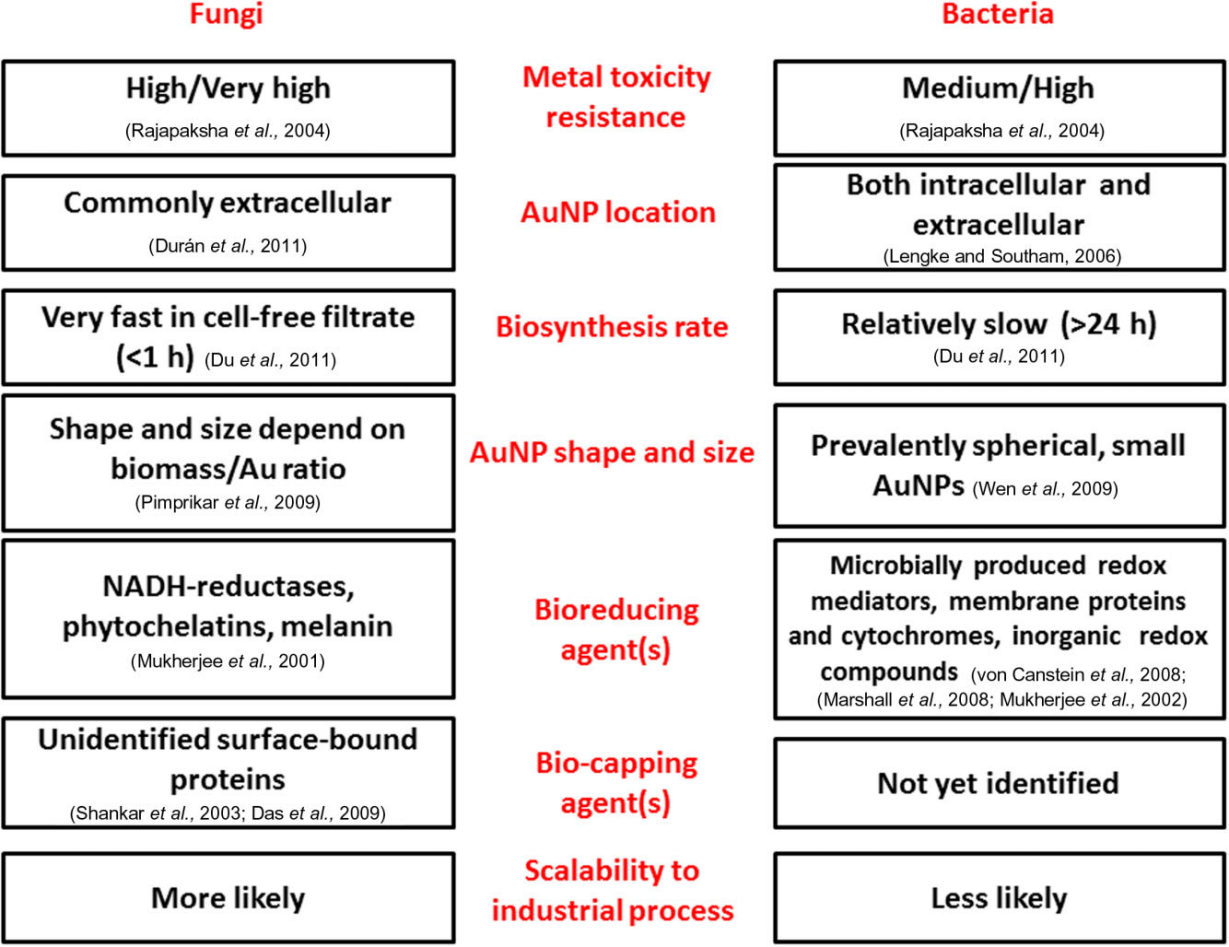
AuNP biosynthesis in fungi vs. bacteria.

**Figure 2:**
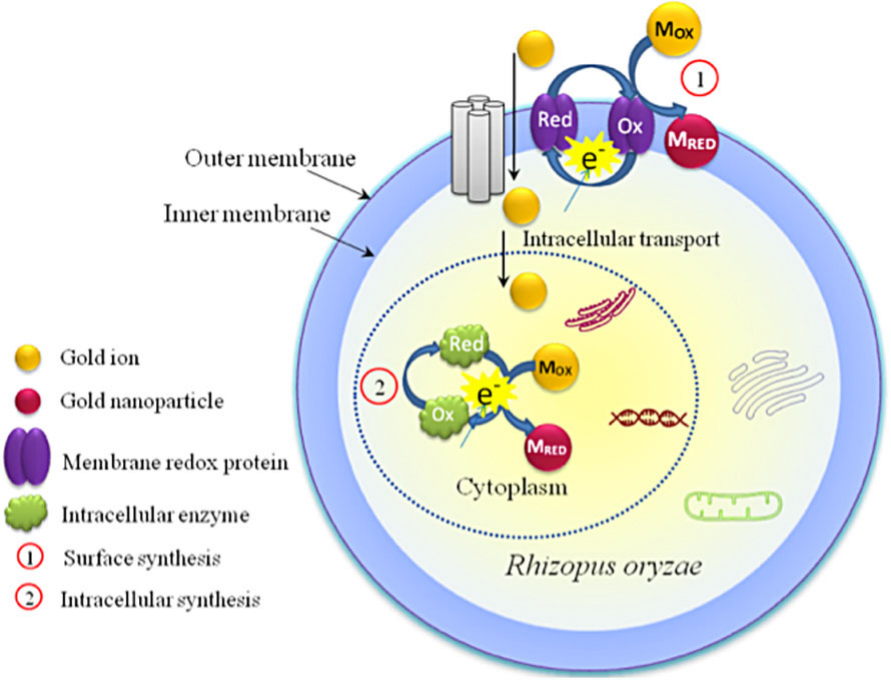
Schematic diagram of a proposed mechanism of Au biomineralization in *Rhizopus oryzae* (Reproduced with permission from Das *et al*., 2012b).

AuNP formation can occur either in the intracellular or extracellular space (Fig. 1, Fig. 2). Extracellular AuNP formation is commonly reported for fungi when Au^3+^ ions are trapped and reduced by proteins in the cell wall. Previous work with the fungus *Verticillium sp*. ruled out the possibility that reduced sugars in the cell wall are responsible for the reduction of Au^3+^ ions and suggested adsorption of AuCl^4-^ ions on the cell-wall enzymes by electrostatic interaction with positively charged groups (e.g. lysine) (Mukherjee *et al*., 2002; Durán *et al*., 2011). In the case of intracellular AuNP formation, Au^3+^ ions diffuse through the cell membrane and are reduced by cystolic redox mediators (Das *et al*., 2012b). However, it is unclear whether the diffusion of the Au^3+^ ions through the membrane occurs via active bioaccumulation or passive biosorption. The latter might be caused by Au^3+^ ions toxicity, which increases the porosity of the cellular membrane. Another recent study reports the production of intracellular AuNPs in metal-tolerant *Aspergillus fumigatus* and *Aspergillus flavus*. The intracellular AuNPs have an average diameter of 22 ± 2 nm, slightly larger than those observed in the extracellular space. The enzymatic reduction mechanism of Au^3+^ is essentially the same for intracellular and extracellular AuNPs (Gupta and Bector, 2013).

In viable cells, Au ions are reduced by nicotinamide adenine dinucleotide (NADH)/nicotinamide adenine dinucleotide phosphate (NADPH) oxidoreductases either in the cell surface or in the cytoplasm. X-ray photoelectron spectroscopy of *R. oryzae* showed that the Au^+^ concentration increases at the beginning of the biosynthetic process, then decreases, while the concentration of Au^0^ increased, demonstrating that Au^3+^ ions are first reduced to Au^+^ and then to Au^0^. The appearance of methylated Au^3+^ was proposed as an additional defence mechanism against metal toxicity (Das *et al*., 2012b).

The SDS-PAGE analysis of whole proteins from *R. oryzae* exposed to Au^3+^ showed up-regulated proteins similar to those observed in the mycorrhizal fungus *Glomus intraradices* exposed to Cd, Cu and Zn (Waschke *et al*., 2006). These results confirm that metals induce oxidative stress response genes. In *R. oryzae*, the up-regulation of those genes occurs at sub-toxic Au concentrations, while at higher concentrations, metal toxicity inhibits growth and protein expression (Das *et al*., 2012b).

*Rhizopus oryzae* shows evidence of intracellular reduction at high Au^3+^ concentrations, which may be associated with Au toxicity. High resolution transmission electron microscopy of the fungus *R. oryzae* incubated with Au^3+^ ions revealed the presence of electron dense particles in the cell-wall and cytoplasmic regions, suggesting that these regions are responsible for the reduction of Au^3+^ to Au^0^ (Das *et al*., 2011a).

Investigation on AuNPs biosynthesis by the soluble protein extract of fungi *Fusarium oxysporum* showed that NADH-dependent reductases are involved in the bioreduction process (Mukherjee *et al*., 2002). However, the specific protein(s) involved in Au reduction have not been yet identified. The appearance of a Fourier transform infrared spectroscopy (FTIR) peak at 1735 cm^−1^ in viable *Phanerochaete chrysosporium* biomass after interaction with the Au^3+^ ions might be relateed to the involvement of aromatic aminoacids like tyrosine and tryptophan in the reduction of Au^3+^ ions (Sanghi *et al*., 2011).

Other mechanisms for fungal AuNP biosynthesis have been proposed. The fungal pathogen *Candida albicans* is capable of synthesizing phytochelatins, which are made of chain links of glucose, cysteine and glycine ((c-Glu-Cys) n-Gly) by the transpeptidation reaction of c-Glu-Cys dipeptide from a succession of glutathione molecules. In the presence of glutathione, metal ions, including Au, trigger phytochelatin synthesis, in which Au^3+^ ions get reduced to AuNPs, which are then capped by glutathione (Chauhan *et al*., 2011).

AuNP synthesis has been detected by UV-visible spectrophotometry in heat-denatured cell-free filtrate of *Sclerotium rolfsii* in the presence of co-enzyme NADPH and 1 mM of Au^3+^. This indicates the involvement of thermostable NADPH-dependent enzymes in the AuNP biosynthesis process (Narayanan and Sakthivel, 2011a). Cyclic voltammetry analysis showed that NADH produced from the fermentation of lactate by *Hansenula anomala* reduces Au^3+^ to AuNPs, despite AuNPs being rapidly reoxidized in lack of exogenous capping agents (Kumar *et al*., 2011).

The involvement of biosynthetic redox mediators in the fungal biosynthesis of AuNPs has been observed in *Yarrowia lipolytica*. These fungi secrete melanin (Apte *et al*., 2013), which interestingly appears to reduce Au^3+^ to AuNPs. However, experiments with melanin mutant and melanin-complemented strains have not been reported. Therefore, it is not certain if reduction by melanin is a plausible pathway *in vivo*. Further experiments from the same research group showed that melanin could mediate the reduction of metal salts to their elemental forms as nanostructures. Melanin is secreted in a phenol form and reduces Au^3+^ to Au^0^, while being oxidized to its quinone form (Apte *et al*., 2013).

### Capping process

Small Au^0^ nanocrystals are unstable, and for this reason, fungi use extracellular proteins as capping agents to minimize AuNP aggregation and thus stabilize the nanocrystal. This process is analogous to the dispersion of AuNPs through chemical agents and results in the formation of dispersed AuNP with a broad particle size distribution. Full control of capping step in the AuNP biosynthesis process might result in the production of AuNP with narrow size and shape distribution that can be readily used in biomedical and industrial applications.

FTIR is the most commonly used method for the analysis of the capping and stabilizing ligands. Since fungi secrete a variety of enzymes and proteins, the specific organic molecule that acts as capping agent cannot be detected. However, the presence of organic molecules in the synthesized nanoparticles can be determined using FTIR. Additionally, the slight shift in the peaks of these functional groups to lower frequencies, which indicate that they might be involved in interactions with another group, thus confirming the capping mechanism. For example, possible involvement of phosphate bonds in AuNP capping was found in AuNPs synthesized by viable *R. oryzae* biomass by the shifting of FTIR bands from 1034 cm^−1^ to 1025 cm^−1^ corresponding to C-N stretching mode, when compared with the pristine biomass control bands (Das *et al*., 2009). The phosphate peak was observed to shift from 1033.6 to 1025.1 cm^−1^. This study also revealed the presence of amide I, II and III groups and the disappearance of carboxyl groups in the mycelia, which suggests the involvement of polypeptides in the biosynthetic mechanism (Das *et al*., 2009). The amide I, II and III peaks shifted from 1652.9, 1550 and 1379 cm^−1^ to 1635.5, 1544 and 1371.2 cm^−1^, respectively, indicating their involvement in capping process. Involvement of proteins in the capping of AuNPs was also indicated in the FTIR analysis of AuNPs synthesized by *Aspergillus oryzae* var. *viridis* by the observation of absorption bands at 1660 and 1530 cm^−1^, which corresponds to amide I and II bands, respectively (Binupriya *et al*., 2010b), which were also observed in AuNPs produced by *Colletotrichum* sp. by absorption bands at 1658, 1543 and 1240 cm^−1^, which corresponds to amide I, II and III bands respectively (Shankar *et al*., 2003).

AuNPs produced by both *Penicillium brevicompactum* (Mishra *et al*., 2011) and *Penicilium rugulosum* (Mishra *et al*., 2012b) showed a broad peak around 3100–3350 cm^−1^, which may correspond to stretching vibrations of amine (NH) or hydroxide (OH) groups. AuNPs synthesized by *Alternaria alternata* revealed absorption bands at 3,430 and 2,920 cm^−1^, which indicates the presence of O-H and aldehydic C-H stretching (Sarkar *et al*., 2012).

Two bands at 1383 and 1112 cm^−1^ detected by FTIR of freeze-dried AuNPs synthesized by *Aspergillus niger* can be assigned to the C-N stretching vibrations of aromatic and aliphatic amines (Bhambure *et al*., 2009). These amines were also detected in AuNPs synthesized by *P. chrysosporium* with absorption bands at 1367 and 1029 cm^−1^ (Sanghi *et al*., 2011). AuNPs produced by *P. brevicompactum* revealed peaks at 2970 cm^−1^, which suggests the involvement of carboxylic and phenolic groups (Mishra *et al*., 2011). FTIR of AuNPs produced by *A. alternata* showed an intense band at 1425 and 874 cm^−1^, indicating a C–H in plane deformation with aromatic ring stretching (Sarkar *et al*., 2012). The appearance of bands at 2854 and 1737 cm^−1^ from the same study corresponds to aromatic C–H anti-symmetric stretching vibration and C = O stretching vibration. These results suggest that proteins bind to AuNPs either through free amine, carboxyl or phosphate groups for stabilization (Sarkar *et al*., 2012). This amine linkage to AuNPs was also detected by the change in the absorption bands related to N atoms detected in AuNPs synthesized by *P. chrysosporium*. Furthermore, the aromatic amino acids tyrosine, tryptophan and sulfur containing the amino acids cysteine and methionine were found to be associated with AuNPs, while the disappearance of the –SH stretching band after interaction with Au ions indicates the formation of Au–S bonds (Sanghi *et al*., 2011). Three putative-capping proteins with a molecular weight of about 100, 25 and 19 kDa from AuNPs synthesized by *F. oxysporum*, were identified as plasma membrane ATPase, 3-glucan-binding protein and glyceraldehyde-3-phosphate dehydrogenase (Zhang *et al*., 2011). The main FTIR results are summarized in Table 3. While FTIR results support the conclusion that proteins are involved in AuNPs capping and stabilization, the identification of the actual capping agent warrants further research.

**Table 3:** FTIR characterization of AuNPs capping and stabilizing agents in various fungal species

Species	Main FTIR peaks that shift following AuNP formation (cm^−1^)	Groups	Putative biomolecule	Reference
*Rhizopus oryzae*	1652.9, 1550 and 1379	Amide I, II and III	Surface-bound protein	Das *et al*., 2009
*Aspergillus oryzae* var. *viridis*	1660 and 1530	Amide I and II	Proteins (through free carboxylate groups)	Binupriya *et al*., 2010a
*Colletotrichum* sp.	1658, 1543 and 1240	Amide I, II and III	Proteins	Shankar *et al*., 2003
*Penicillium brevicompactum*	3100–3350 (broad peak)	NH or OH		Mishra *et al*., 2011
*Aspergillus niger*	1383 and 1112	Aromatic and aliphatic C-N	Proteins	Bhambure *et al*., 2009
*Phanerochaete chrysosporium*	1367 and 1029	Aromatic and aliphatic C-N	Proteins	Sanghi *et al*., 2011
*Alternaria alternata*	1625, 1425, 874 and 1240	Amide I,C-H deformation, C-H aromatic, and Amide III	Proteins	Sarkar *et al*., 2012

### Comparison between bacterial vs. fungal biosynthesis of AuNPs

Comparing the efficiencies of AuNPs biosynthetic processes in different microorganisms is not straightforward (Fig. 1). The biosynthetic process in itself depends on various factors like temperature, pH, metal ion concentration and inoculum age, which differ widely across published work (Dhillon *et al*., 2012). Furthermore, morphological characterization (i.e. size, shape, distribution, crystallinity and growth kinetics) of the AuNPs is not reported routinely.

Rapid biosynthesis has been achieved in *Penicillium* sp. 1-208 cell filtrate, where near quantitative bioreduction and AuNP synthesis was obtained in just under 5 min. It is suggested that fungi secrete a large amount of extracellular enzymes in a relatively pure state, free from other cellular proteins. Suspension of fungal biomass in non-growth medium for 2 days prior to cell filtration increases the concentration of extracellular redox proteins (Du *et al*., 2011). The Au^3+^ bioreduction using viable fungal biomass of the same strain required about 8 h. Au^3+^ complete bioreduction time reported in Table 2 range from 8 h in *Penicillium* sp. 1-208 (Du *et al*., 2011) to 168 h in *Cylindrocladium floridanum* (Narayanan and Sakthivel, 2011b). Much shorter reaction times are reported for AgNPs biosynthesis under light conditions, as fungal biomass catalyze light-driven reduction of Ag^+^ to Ag^0^ (Wei *et al*., 2014). However, initial appearance of AuNPs in viable *Fusarium semitectum* biomass after 4 h (Sawle *et al*., 2008) and even in a few minutes in *Aspergillus terreus* biomass (Priyadarshini *et al*., 2014). Rapid Au^3+^ bioreduction kinetics in cell-free filtrate of *Trichoderma viride* (Mishra *et al*., 2014) and *Botrytis cinerea* supernatant (Castro *et al*., 2014) confirms that the majority of Au-reducing proteins are secreted in the extracellular space and not associated with cell surface or cytoplasm.

Fungi produce AuNPs with a broader size distribution than bacteria. As the molecular biology of bacteria is much more established, they are currently preferred for AuNPs over fungi and other eukaryotic organisms (Kalathil *et al*., 2011). A systematic study on metal nanoparticle biosynthesis in about 200 fungal genera showed that only two species, *Verticillium* sp. and *F. oxysporum*, produced metal NPs (Du *et al*., 2011). Nevertheless, metallic NPs biosynthesis has been reported (Table 3) since then, as other fungal species have been investigated. Fungi show promises for industrial AuNP biosynthesis because of their easy handling (Binupriya *et al*., 2010b), larger protein secretion than bacteria, high biomass yield (Du *et al*., 2011), and accessibility for a scale up (Narayanan and Sakthivel, 2012b).

One of the major concerns against the use of biosynthetic AuNPs in biomedical applications is that these AuNPs carry proteins of fungal or bacterial origins as capping agents. Our immune system can recognize these proteins and promote an immune response similar to that observed during a fungal or bacterial infection. However, such immune response is an unwanted side-effect if AuNPs are used as drug carrier (Jain *et al*., 2012). Recent studies in immunology have shown that many microorganisms glycosylate their membrane proteins to reduce or even suppress the immune response of the host (Rudd *et al*., 2001). Glycosylation, either via microbially produced or host-produced oligosaccharides avoid recognition of the pathogen's proteins by the host-binding proteins, thus reducing or suppressing the innate immune response. For example, the rice blast fungus suppresses the rice immune response (Chen *et al*., 2014). Considering that glycosylation is much more prominent in fungal than in bacterial protein synthesis, fungal proteins involved in the capping and stabilization of AuNPs are also less likely to induce an immune response (Li and d'Anjou, 2009). As the study of microbiota exoglycome and their implications in the immune response are merely mentioned here, the interested reader should refer to a recent comprehensive review in this area.

### Design of a bioreactor for AuNPs biosynthesis

AuNPs biosynthesis processes and nanoparticle biosynthesis in general are still carried out at laboratory scale using suspended fungal biomass or their cell-free extracts. Scale up of AuNPs biosynthesis will require bioreactors where the fungal biomass or the protein extracts are conveniently immobilized in a thin layer to minimize diffusional limitation as well as the time needed for biosynthesis. Furthermore, AuNPs should be easily recovered in the outlet after the bioreduction process to minimize downstream processing costs. Currently, there is no available report on fungal biomass reactors for AuNP biosynthesis. However, several bioreactors have been already described where fungal biomass serves as a catalyst for any given bioprocess (Ciudad *et al*., 2011; Djelal and Amrane, 2013; Yang *et al*., 2013). For example, *P. chrysosporium* was grown as thick biofilm (∼ 0.4–1.0 mm) on polysulfonic and tubular ceramic membranes (Sheldon and Small, 2005). More recently, high concentration of *Rhizopus oligosporus* fungal biomass was grown in a 2.5 L batch airlift bioreactor (Nitayavardhana *et al*., 2013). Contaminant removal from water was achieved using active *Trametes versicolor* biomass in a 10 L fluidized bed bioreactor (Morato *et al*., 2013). The design and the operative conditions of a bioreactor for AuNP biosynthesis largely depends on the concentration and location of nanoparticles (intracellular vs. extracellular) and the concentration of fungal biomass. Extracellular AuNPs must be purified from planktonic biomass, cell debris and cell secretome. Conventional stirred tank reactors such as those proposed to synthesize extracellular chitinases (Barghini *et al*., 2013) provide standardized operation and easy scale up but the downstream processing and the separation of biosynthetic AuNPs might prove less cost-effective. Other methods for metal nanoparticle purification include dialysis (Aqil *et al*., 2008) gel filtration, evaporation (Limayem *et al*., 2004), ion exchange (Zhang *et al*., 2010), centrifugation (Wulandari *et al*., 2008) and diafiltration (Sweeney *et al*., 2006). A possible strategy for nanoparticle purification could employ concentrated poly (ε-caprolactone) nanocapsules using crossflow filtration, followed by purification using dialysis (Limayem *et al*., 2004). Crossflow microfiltration is suitable for the processing of large nanoparticulate suspension volume, as the membrane surface can be increased according to the volume to be treated.

If AuNPs are transported inside the cell wall, then additional steps for their recovery, such as cell lysis will be needed, similar to those employed in the recovery of intracellular metabolites produced at industrial scale. However, these additional steps might render the process not feasible from an economic standpoint.

## Conclusions and future perspectives

Green synthesis of AuNPs has attracted attention because of their reduced environmental toxicity. Bacterial and fungal biosynthesis of AuNPs offer similar process performance. However, fungi might be a better candidate for scale up because of the large amount of extracellular redox enzymes that they secrete. At a fundamental research level, poorly characterized fungi from diversity hotspots in tropical and equatorial environment may harbour novel pathways for metal nanoparticle synthesis. Finally, capping agents of fungal origin are less likely to stimulate an immune response like that induced by bacterial proteins.

Recent studies on extracellular synthesis of AuNPs in fungi suggest that the enzymatic machinery required for their biosynthesis is similar to that used for metal detoxification. Intracellular AuNP biosynthesis is reported to occur when there is a high Au^3+^ concentration available and when the membrane integrity is compromised to allow for Au^3+^ ions diffusion within the cell. However, there is no evidence available yet showing that fungi use biosynthetic nanoparticles for their metabolism.

Au bioreduction occurs in two main steps: (i) Au^3+^ is reduced to Au^+^ and (ii) Au^+^ is reduced to Au^0^. The frequent observation of one-step reduction of Au^3+^ to Au^0^ may be due to the short life of the Au^+^ form at ambient temperature. Controlled experimental conditions are needed to validate the Au bioreduction mechanism. Both proteins and other secondary metabolites may serve as capping and stabilizing agents for AuNP biosynthesis. Currently, only a handful of biomolecules involved in bioreduction and capping of AuNPs has been reported. Further research is needed to identify other species involved in the process.

The most desired candidates for large-scale AuNPs biosynthesis are those fungi that secrete large amount of extracellular enzymes and metabolites, resulting in the formation of well-dispersed AuNPs in the extracellular space. Among other candidates, the filamentous fungi *Aspergillus* and *Trichoderma* sp. produce numerous extracellular enzymes and metabolites of industrial interest (Meyer *et al*., 2011) and their genetics is reasonably well known. Therefore, they are excellent model organisms for in-depth genetic and biochemical investigation of bioreduction process.

Particle size distribution is probably the most important parameter, as small nanoparticles are very important as drug carriers and catalysts. In a NaBH_4_ chemical reduction process, highly monodispersed spherical nanoparticles with diameter < 5 nm diameter were obtained through accurate control of thiol-containing polymers that cap AuNPs, preventing their aggregation (Wang *et al*., 2007). In a physical bottom-up process employing microwaves, the use of high microwave power favours homogeneous nucleation, thus decreasing the AuNP size while increasing their uniformity. Monodispersed spherical AuNPs of 12 ± 1 nm diameter were produced (Seol *et al*., 2011). These results imply a higher control over the particle size and shape in chemical synthesis techniques.

Currently, AuNPs biosynthesis process in fungi does not compare favourably with the particle size distribution attainable in advanced physicochemical processes. Transmission electron microscopy-calculated particle size distributions are reported only for a small number of fungal species (Table 2). The particle size distributions reported in these studies is generally broader (10–40 nm) than that observed in physicochemical processes. Furthermore, the particle size distribution depends on the fungal catalyst used, the gold/biomass ratio and other process conditions. As microorganisms produce a variety of proteins that bind to nanoparticles of different sizes (Lundqvist *et al*., 2008), genetic and metabolic control of the nanoparticle production in viable fungal biomass is crucial for the reproducibility of the AuNP biosynthesis process.

The synthesis of highly stable AuNPs of defined shape and particle size distribution would require the optimization of numerous parameters, including pH, biomass/Au^3+^ ratio, and operative conditions of the bioreactor. However, with a proper optimization of these parameters, the above issue can be addressed. The slow kinetics of AuNP formation may be addressed by using cellular filtrate instead of viable biomass. If viable biomass is used, high surface bioreactors are needed to speed up the biosynthetic process.
